# Screening for psychoaffective disorders in night-shift nurses : a cross sectional study

**DOI:** 10.1192/j.eurpsy.2025.1032

**Published:** 2025-08-26

**Authors:** N. Rmadi, A. Hrairi, F. Bouzid, A. Kchaou, N. Kotti, M. Hajjaji, K. Jmal Hammami

**Affiliations:** 1 Occupational medicine department, Hedi chaker university hospital, University of Sfax; 2 Family medicine department, University of Sfax, Sfax, Tunisia

## Abstract

**Introduction:**

Night shift work disrupts the natural circadian rhythms, leading to significant sleep disturbances that can adversely affect mental health. Nurses working these shifts often report higher levels of stress, anxiety, and depression compared to their day-shift counterparts.

**Objectives:**

This study aims to assess the prevalence of psycho-affective disorders among night-shift nurses.

**Methods:**

Data were collected from a cross sectional study in the university hospitals of Sfax. We used a questionnaire exploring socio-demographic, professional data and pathological history. We evaluated absenteeism based on the number of days absent over the past year. Psychoaffective disorders were screened using the validated Arabic version of the Depression, Anxiety, and Stress Scale: DASS-21.

**Results:**

Our study included 114 nurses, with 65% being female. The mean age was 33.8 ans ± 7 years. Severe to very severe symptoms of depression, anxiety, and stress were found in 14%, 21%, and 18% of our participants, respectively. Among our workers, 58% reported being moderately satisfied at work. During the last 12 months, 68 nurses (59.6%) were absent for an average of 27.21 days ± 72.73 days. Significant associations were found between job satisfaction and severity of depression (p= 0.000), anxiety (p=0.027) and stress (p = 0.000). Absenteeism was significantly associated to depression (p=0.009, r=0.24), anxiety (p=0.014, r=0.23) and stress (p=0.35, r=0.19).

**Conclusions:**

Psychoaffective disorders are common among paramedical staff working shifts in hospitals. It is essential to conduct screening consultations in occupational medicine to identify these disorders early on.

**Disclosure of Interest:**

None Declared

